# Changes in the Salivary Metabolic Profile of Generalized Periodontitis Patients after Non-surgical Periodontal Therapy: A Metabolomic Analysis Using Nuclear Magnetic Resonance Spectroscopy

**DOI:** 10.3390/jcm9123977

**Published:** 2020-12-08

**Authors:** Filippo Citterio, Federica Romano, Gaia Meoni, Giovanni Iaderosa, Silvia Grossi, Alberto Sobrero, Francesca Dego, Matteo Corana, Giovanni Nicolao Berta, Leonardo Tenori, Mario Aimetti

**Affiliations:** 1 Department of Surgical Sciences, C.I.R. Dental School, Section of Periodontology, University of Turin, 10126 Turin, Italy; federica.romano@unito.it (F.R.); g.iaderosa@hotmail.it (G.I.); silvia.grossi95@hotmail.it (S.G.); albertosobrero@gmail.com (A.S.); degofrancesca@gmail.com (F.D.); matteo.corana@gmail.com (M.C.); mario.aimetti@unito.it (M.A.); 2 Giotto Biotech S.R.L, Via Madonna del Piano, 6, 50019 Sesto F.no, Florence, Italy or meoni@cerm.unifi.it; 3 Department of Clinical and Biological Sciences, University of Turin, San Luigi Gonzaga, 10043 Orbassano, Italy; giovanni.berta@unito.it; 4 Magnetic Resonance Center (CERM), University of Florence, 50019 Sesto F.no, Florence, Italy; tenori@cerm.unifi.it; 5 Department of Chemistry, University of Florence, 50019 Sesto F.no, Florence, Italy

**Keywords:** diagnosis, metabolomics, periodontal therapy, periodontitis, saliva

## Abstract

Pattern analysis of the salivary metabolic profile has been proven accurate in discriminating between generalized periodontitis (GP) patients and healthy individuals (HI), as this disease modifies the salivary concentrations of specific metabolites. Due to the scarcity of data from previous studies, this study aimed to evaluate if non-surgical periodontal therapy (NST) could affect the metabolomic profile in GP patients’ saliva and if it compares to that of HI. Unstimulated salivary samples were collected from 11 HI and 12 GP patients before and 3 months after NST. Nuclear Magnetic Resonance (NMR) spectroscopy, followed by a supervised multivariate statistical approach on entire saliva spectra and partial least square (PLS) discriminant analysis, were performed to obtain metabolic profiles. In the GP group, periodontal treatment improved all clinical parameters, but not all the diseased sites were eradicated. PLS revealed an accuracy of 100% in distinguishing between metabolic profiles of GP patients before and after NST. Orthogonal projection to latent structure was able to discriminate between the three groups of subjects with an accuracy of 85.6%. However, the post-NST metabolic profile of GP patients could not be completely assimilated to that of HI. Although NST may produce significant changes in the metabolic profile, GP patients maintained a distinctive fingerprint compared to HI.

## 1. Introduction

Periodontitis is a chronic inflammatory disease that leads to the loss of periodontal attachment, resorption of alveolar bone, and, if left untreated, to tooth loss. It is spread worldwide and affects a large proportion of adult subjects [1]. Periodontitis is caused by a dysbiosis between the host and the bacterial biofilm that colonizes the dental surfaces. Conventional non-surgical periodontal therapy (NST) consists of oral hygiene instruction, patient motivation and the mechanical removal of supra- and sub-gingival periodontal biofilm deposits [2]. The mechanical removal of the biofilm is effective in improving clinical parameters, reducing inflammation and restoring the host-microbial symbiotic state [3,4]. At present, the diagnosis of periodontitis (together with the assessment of treatment outcome) is primarily based on clinical and radiographic examination [5]. 

Gingival crevicular fluid (GCF) and saliva have been investigated in an attempt to provide a detailed understanding of the molecular changes associated with periodontal tissue destruction [6]. Saliva has already proved to be a viable biofluid for the identification of alterations occurring in systemic and oral health status [7]. It is composed of a high variety of locally synthetized and systemically derived molecules involved in various metabolic processes and it is influenced by the interactions that occur in between the patient, the microbiome and environmental factors [8,9]. Importantly, the salivary metabolome is easily accessible and has revealed specific metabolomics signatures for numerous oral or systemic diseases [10]. NMR-based metabolomics has provided significant information on oral conditions, such as dental caries [11], oral cancer [12], and Sjögren’s syndrome [13], as well as on systemic conditions, such as dementia [14], neurodegenerative disorders [15], cardiovascular diseases [16,17] and viral infections [18].

As a consequence, metabolomics has recently emerged as a relevant approach for the investigation of the phases (i.e., onset, progression, treatment response) of periodontal diseases [19]. It deals with the high-throughput identification and quantification of metabolites in biological fluids by means of Nuclear Magnetic Resonance (NMR) and Mass Spectrometry (MS) [20,21,22]. Several studies have demonstrated that the metabolic signature of saliva samples collected from chronic periodontitis patients is different from that of healthy controls, reaching discrimination accuracies up to 84% [1,23,24]. Metabolites defining the periodontal status are associated with either bacterial metabolic activity (lactate, pyruvate, formate) [23] or tissue degradation (proline, phenylalanine, tyrosine) [25,26], or even host immune response (valine, isoleucine) [27]. 

It has also been proposed that salivary biomarkers could be useful for monitoring the response to NST, in terms of changes in salivary content of pro-inflammatory cytokines and proteinases [28,29,30,31].

This study aimed to determine whether the salivary metabolic profile of patients with generalized severe periodontitis changes after NST, and if the metabolomic profile of treated patients compares to that of periodontally healthy patients.

## 2. Materials and Methods

### 2.1. Study Design and Population

Participants were consecutively recruited at the C.I.R. Dental School, University of Turin (Italy) during the period from April to September 2018. The protocol of the study was approved by the Institutional Ethical Review Board (Protocol n° 1503/2016) and the study was conducted in accordance with the Helsinki Declaration (as revised in 2002). Written informed consent was obtained from all participants.

Patients were included in the study if they presented with generalized stage III periodontitis (GP) with at least 20 natural teeth, and with ≥30% of teeth with a clinical attachment level (CAL) of ≥5 mm and the presence of bleeding on probing (BoP). No more than four teeth should have been lost due to periodontitis and the complexity factors defining the need for a complex rehabilitation should not have been satisfied [5,32]. Furthermore, healthy individuals (HI) that had at least 20 natural teeth, no interdental CAL loss at >1 non-adjacent tooth, probing depth (PD) ≤ 3 mm, ≤10% of sites with BoP and no radiographic evidence of bone loss [33] were selected among the undergraduate students of the C.I.R. Dental School.

The subjects in both groups were non-smokers. None had received periodontal treatment over the previous 6 months or antibiotic treatment within the previous 3 months. 

Individuals suffering from systemic conditions including cardiovascular diseases, diabetes and other systemic disorders that could affect the periodontal tissues or could impact the metabolic profile were excluded. Subjects using medications with a confirmed side effect on periodontal tissues such as phenytoin, cyclosporine, anti-inflammatory drugs, or calcium channel blockers were also excluded. None of the women were pregnant or in the lactation period.

### 2.2. Clinical Examination and Periodontal Treatment

At baseline, one previously calibrated periodontist (95.3% intra-examiner concordance) performed a complete periodontal examination on all participants. Periodontal biometric parameters were recorded at six sites per tooth (excluding third molars) using a North Carolina periodontal probe (Hu-Friedy, IL, USA). At the same sites, the presence of plaque [34] and BoP were recorded dichotomously. The number and percentage of PDs ≥ 4 mm and PDs ≥ 6 mm, the number of teeth, full-mouth plaque score (FMPS) and full-mouth bleeding score (FMBS) were also recorded.

After clinical and radiographic examination, GP patients underwent a session of supragingival scaling and received oral hygiene instructions. Subgingival scaling and root planing (SRP) were performed by a single trained clinician on a quadrant-wise protocol using hand instruments (Gracey curettes, Hu-Friedy) together with a magnetostrictive device (Cavitron Select, Dentsply, York, PA, USA). NST was completed within 28 days and afterwards patients were recalled every month for supragingival prophylaxis and oral hygiene reinforcement. The periodontal parameters described above were recorded again 3 months after the completion of NST. HI did not receive any treatment but oral hygiene instructions were reinforced.

### 2.3. Saliva Sampling Collection

To prevent blood contamination, saliva samples were collected between 9:00 and 11:00 a.m. on the day following the clinical examination. The participants had to avoid eating, drinking, rinsing with mouthwashes and toothbrushing for at least 1 h before saliva collection. Each subject was recommended to allow saliva accumulation in the mouth, and then let the saliva drain into a sterile graduated polypropylene tube until a minimum of 1.0 mL of unstimulated saliva was collected and immediately frozen at −80 °C. In the GP group, saliva sampling was repeated 3 months after NST.

### 2.4. Salivary Metabolomics Profiling

The frozen saliva samples were thawed at room temperature and were centrifuged (5000× *g* for 30 min at 4 °C) to remove debris and to precipitate cells. A total of 300 μL of sodium phosphate buffer [70 mM Na_2_HPO_4_ (Merck KGaA, Darmstadt, Germany); 20% (*v*/*v*) ^2^H_2_O; 6.15 mM NaN_3_ (Merck KGaA, Darmstadt, Germany); 6.64 mM sodium trimethylsilyl [2,2,3,3-_2_H_4_] propionate (TMSP; Merck KGaA, Darmstadt, Germany); pH 7.4) was added to 300 μL of the supernatant, and the mixture was homogenized by vortexing for 30 s. An aliquot of 450 μL of this mixture was transferred into a 4.25 mm NMR tube (Bruker Biospin srl, Milan, Italy) for analysis. Monodimensional _1_H NMR spectra were acquired using a standard pulse sequence (noesygpprd.comp, Bruker Biospin, Milan, Italy) using 128 scans, receiver gain 11.3, 65,536 data points, a spectral width of 12,019 Hz, a relaxation time of 4 s and a mixing time of 0.1 s working at 300 K using a Bruker 600 MHz spectrometer. Transformed spectra were automatically corrected for phase and baseline distortions and calibrated using RMN processing software (Tospin 2.1, Bruker BioSpin srl, Milan, Italy). Spectra were aligned by calibrating the TMSP peak at 0.00 ppm and segmented into 0.02 ppm chemical shift bins (water resonance region from 4.3 ppm to 6.5 ppm was excluded from the bins), and the corresponding spectral areas were integrated using AMIX software (Bruker BioSpin, version 3.8.4) [35,36]. The total area was calculated on the bins and total area normalization was carried out on the data prior to pattern recognition.

### 2.5. Statistical Analysis

All data analyses were performed using the R statistical package. The significance of changes in clinical data with time in the GP group was determined using the paired *t*-test. An unpaired *t*-test was applied to test the differences between periodontitis and healthy subjects. 

Multivariate partial least square (MPLS) analysis was applied to compare the metabolic profile of GP patients before and after NST [37], moreover, Orthogonal Projections to Latent Structures-Discriminant Analysis (OPLS-DA) was used to compare the profile of the HI group with the GP group at baseline and the GP group after non-surgical periodontal therapy. The accuracy was assessed for all the models employing a Monte Carlo cross-validation scheme. All metabolites were assigned according to the available literature and reference databases (e.g., HMDB, BIOREFCODE) [10,38]. The relative concentration of the various metabolites in the different spectra was calculated by integration of the signal area [39]. Based on the assumption that metabolites are not normally distributed in the salivary samples, the Kruskal–Wallis test was used to infer statistically different metabolites’ levels between HI and GP patients before and after NST, and the Wilcoxon signed-rank test and the U Mann–Whitney test were chosen to ascertain pairwise differences among the two time points (before and after NST) and between HI and GP patients after treatment. False discovery rate (FDR) correction was applied using the Benjamini–Hochberg method, and an adjusted *p*-value < 0.05 was considered statistically significant.

## 3. Results

Saliva samples were collected from 12 GP patients (7 females and 5 males, mean age of 62 ± 4.9 years) and 11 HI (6 females and 5 males, mean age of 23.8 ± 0.4 years). The participants were balanced with respect to gender (*p* > 0.05), but not to age (*p* < 0.05). 

The clinical parameters of HI and GP patients at baseline and after treatment are presented in Table 1. The number of remaining teeth was higher in HI (*p* < 0.005). As expected, the clinical conditions of GP patients were significantly worse than those of HI (*p* < 0.001). At re-evaluation, all GP subjects reported uneventful post-treatment course. Three months after completion of treatment, FMPS and FMBS decreased significantly in the GP group compared to baseline values (*p* < 0.001). Periodontal treatment was also associated with a decrease in the mean percentage of sites with PD ≥ 4 mm and PD ≥ 6 mm (*p* < 0.001), even if they remained higher compared to HI (*p* < 0.001). 

**Figure 1:**
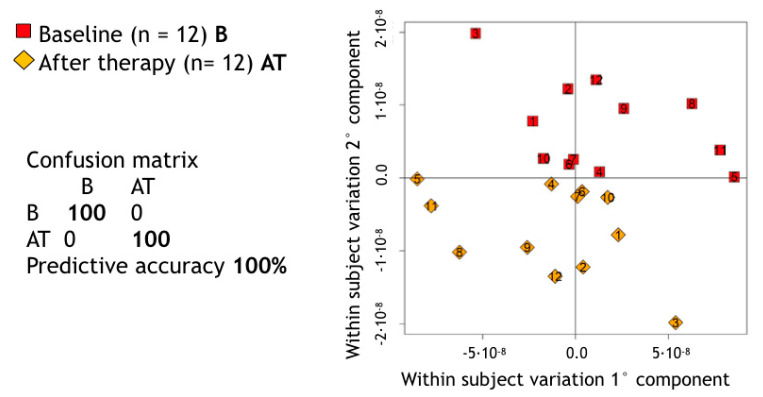
Score plot of Multilevel partial least square (PLS) pairwise discrimination of baseline (B) and after therapy (AT) salivary samples in generalized periodontitis (GP) patients. *p*-value calculated using a premutation test procedure was <0.01 (*p* = 0.0099).

A supervised MPLS pairwise approach allowed us to assess the impact of the NST on the metabolic composition of saliva in GP patients. The predictive accuracy of such a statistical model appeared able to discriminate between the metabolic spectrum of each untreated GP patient and its own counterpart after treatment (predictive accuracy = 100%—Figure 1).

**Figure 2:**
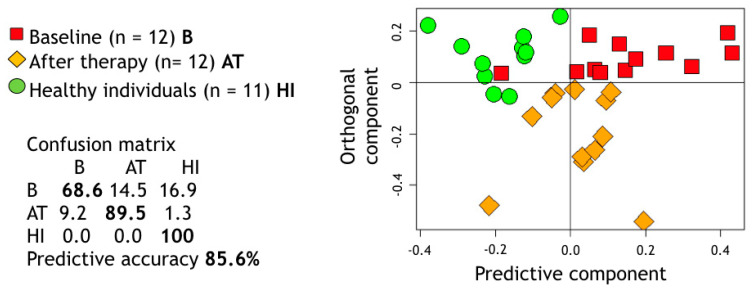
Projection of the proton nuclear magnetic resonance (^1^H NMR) spectral buckets on the two dimensions of the Orthogonal Projections to Latent Structures-Discriminant Analysis (OPLS-DA) considering the subspace for healthy individuals (HI) and generalized periodontitis (GP) patients at two different treatment stages (baseline, B, and after treatment, A)T. The relative confusion-matrix and overall predictive accuracy (calculated following 100 runs of Monte Carlo cross validation) are also reported. *p*-value calculated using a premutation test procedure was <0.01.

Moreover, OPLS-DA was used to assess the shift between the metabolic spectra of saliva from HI and GP patients at baseline and 3 months post treatment and provided an overall discrimination accuracy of 85.6% (Figure 2). As displayed, GP treated patients occupied a portion of metabolic space just in between their own baseline spectra and HI. 

**Figure 3:**
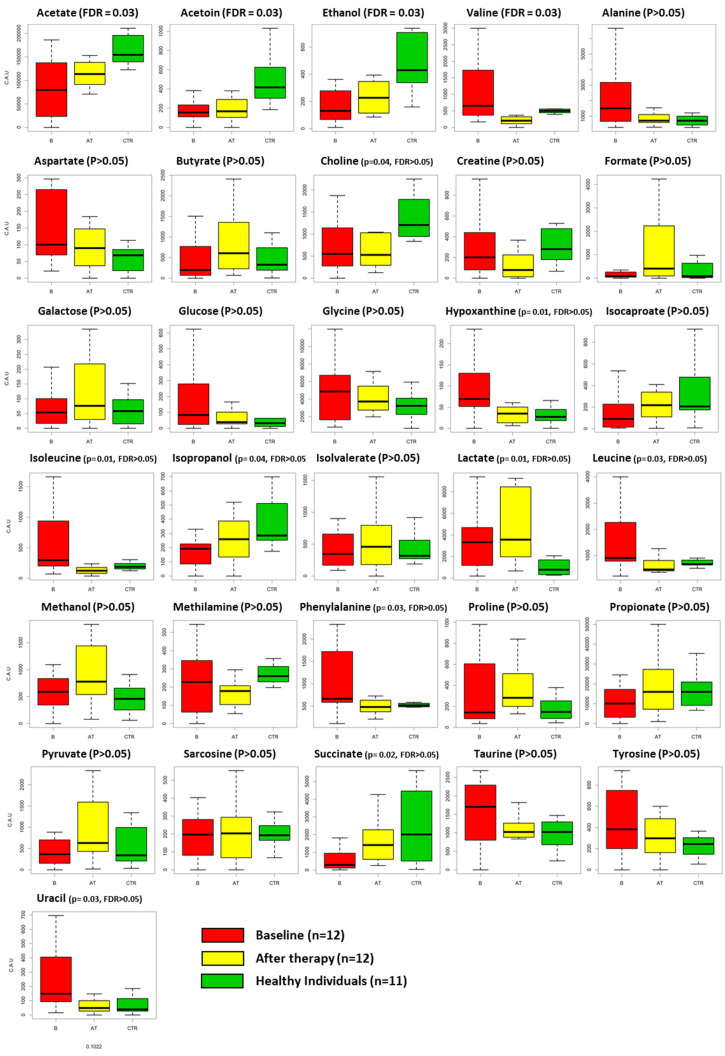
Boxplots representing the fluctuations of each metabolite among the three groups: GP patients at baseline (B, red box), GP patients after non-surgical therapy (AT, yellow box) and HI (healthy individuals, green box). *p*-value and FDR values are also reported. FDR < 0.05 is considered statistically significant.

The changes in individual metabolites among the three groups are reported in Table 2 and depicted in Figure 3.

Kruskal–Wallis enlightened differences among the three groups for the concentrations of acetate (*p* = 0.002), ethanol (*p* = 0.003), succinate (*p* = 0.022), leucine (*p* = 0.035), valine (*p* = 0.005), acetoin (*p* = 0.002), phenylalanine (*p* = 0.036), isoleucine (*p* = 0.012), lactate (*p* = 0.019), choline (*p* = 0.041), hypoxanthine (*p* = 0.010), uracil (*p* = 0.03) and isopropanol (*p* = 0.048). After correction for multiple comparison, acetate, ethanol, valine, and acetoin remained significant (FDR < 0.05). When comparing HI and GP groups after treatment, we observed reduced concentrations of acetate (*p* = 0.004) and lactate (*p* = 0.012) and increased concentrations of ethanol (*p* = 0.036), proline (*p* = 0.016), valine (*p* = 0.009), acetoin (*p* = 0.002), methylamine (*p* = 0.006), lactate (*p* = 0.0011), creatine (*p* = 0.019) and choline (*p* = 0.023) in the saliva of HI. However, none of these differences remained significative after correction (FDR ≥ 0.05). Finally, when comparing the saliva from GP subjects before and after treatment, we observed a reduction in the concentration of leucine (*p* = 0.045), valine (*p* = 0.001), phenylalanine (*p* = 0.031), isoleucine (*p* = 0.014), hypoxanthine (*p* = 0.031) and uracil (*p* = 0.004) and an increased concentration of formate (*p* = 0.016) at the completion of NST. However, only valine maintained an adjusted *p*-value < 0.05 after correction.

## 4. Discussion

The present study was designed to determine whether NST could change the salivary metabolomic profile of patients with generalized periodontitis. 

The MPLS score plot revealed that NST produced a shift in the metabolomic composition of saliva, probably related to the reduced levels of inflammation in the periodontal tissues after treatment and to a significant change within the oral microbiome [40,41]. The accuracy of the multivariate model in discriminating between the metabolomic profile of GP patients before and after treatment was 100%. Together with the metabolomic changes, the periodontal treatment led to a significant improvement in all recorded clinical parameters (*p* < 0.001). This improvement is in line with those reported in previous studies [42]. During the study period, patients were enrolled in a strict recall program for professional debridement and reinforcement of self-performed oral hygiene. This resulted in a low level (<20%) of plaque scores throughout the study period.

Single metabolite fluctuations may be linked to different events occurring in health and disease and to the changes produced by treatment. Interestingly, we identified differences in the concentrations of a variety of metabolites among the three groups, and after correction for multiple comparison acetate, ethanol, valine and acetoin remained statistically significant (FDR < 0.05). On the other hand, the adjusted *p*-value for multiple comparison in pairwise analyses (B vs. AT and HI vs. AT) failed to reach statistical significance for all metabolites except for valine in GP patients before and after treatment. However, due to the limited sample size of the present study, we decided to also discuss the results according to *p*-values before the FDR correction. In this context, we observed that the levels of valine and isoleucine decreased after NST (*p* < 0.005), as they reflect host immune response to the oral microbiome and have already been associated with active periodontal disease [27]. In parallel, uracil decreased as it has been related to BoP, PDs and CAL [43]. Importantly, the reduction in the concentration of hypoxanthine after treatment confirms that NST is able to counterbalance the metabolic alteration occurring at cellular level as a consequence of bacterial insult. In fact, hypoxanthine concentration is related to the bacteria-mediated degradation of purine that occurs during oxidative stress events, which are common at periodontally compromised sites [44]. The increased concentration of formate is associated with a reduction in the number of anaerobic bacteria present in the oral environment. The decreased phenylalanine could be a consequence of reduced host tissue degradation after NST [25,26].

Differences in specific metabolites were also found between healthy subjects and treated periodontitis patients. The lower concentration of lactate and the higher concentration of methylamine may be linked back to bacterial metabolism. The higher level of proline in treated periodontitis patients could be explained by the up-regulation of protease activity found in periodontal disease [39]. The higher level of choline in healthy patients is in agreement with the findings from García-Villaescusa et al. [45], who found a greater concentration of this metabolite in healthy patients compared to gingivitis/early periodontitis subjects. Finally, the lower level of acetate in treated individuals is in contrast with previous evidence [1,46] and difficult to interpret.

The second purpose of this investigation was to test the hypothesis that NST could restore the disease-related alterations in the individual salivary metabotype to a profile similar to that of periodontally healthy patients. Taking into consideration the obvious difficulties in designing a study that would allow us to harvest salivary samples before and after the onset of periodontal disease, we decided to compare the metabolome of post-treatment salivary samples with that of a group of independent HI. Looking at the results of the OPLS-DA analysis, the metabolic profile from treated periodontitis patients (3-month follow-up) occupied an intermediate space of the 3-dimensional plot, between healthy subjects and the samples collected before non-surgical treatment. In agreement with our previous investigation [47], it is not possible to assimilate the metabolic profile of treated periodontitis patients to that of healthy subjects. One possible explanation could be the difference in age between them. Furthermore, it is possible that non-surgical periodontal treatment is not resolutive in all patients [48,49] and additional interventions, i.e., antimicrobials and/or surgical therapy, could be necessary to achieve all treatment goals [50,51]. Indeed, in the present investigation, most of the treated patients displayed some residual pockets and sites with persistent inflammation at the 3-month re-evaluation. The confounding effect of residual diseased sites could be overcome by comparing only pre- and post-treatment metabolic profiles of patients who achieve all treatment goals. Furthermore, 3 months could be too short a period to detect any relevant metabolic change after the completion of NST. Another explanation could be that periodontitis patients still hold a metabolic signature of their individual susceptibility to the disease even after successful NST. This speculation requires further investigation, since it is still unclear whether individual susceptibility to periodontal disease lies in the local immune-inflammatory reactivity, in the microbiome or at the systemic metabolic level [52,53]. In the future, it would be useful to compare the pre- and post-treatment metabolomic profiles of successfully treated patients (≤10% of sites with BoP, no periodontal pockets >4 mm with BoP or no deep periodontal pockets (≥6 mm)) with the salivary metabolome of responders versus non-responders to periodontal treatment, possibly over a longer follow-up period. 

## 5. Conclusions

Within the limitations of the present study, NMR-spectroscopy revealed that periodontitis patients maintained a distinctive metabolic profile compared to healthy individuals even after treatment, even though therapy generated consistent fluctuations in the metabolites’ concentrations. Metabolic analysis could be used to monitor treatment stages and may discriminate between patients with active or previous periodontitis and healthy individuals.

## Figures and Tables

**Table 1:** Demographic and clinical parameters in healthy and periodontitis patients before and after non-surgical periodontal treatment.

	Healthy Individuals (HI)		Generalized Periodontitis (GP) Patients			
			Baseline		After Treatment	
	Mean	SD	Mean	SD	Mean	SD
Age (years)	23.82	0.40	55.25	4.88	55.25	4.88
N. of teeth	27.73	2.33	26.67	1.44	26.67	1.44
N. of sites	166.36	13.97	160.00	8.61	160.00	8.61
N. of PD ≥ 4 mm	2.18	1.25	83.42	33.73	28.58	23.77
% of PD ≥ 4 mm	1.33	0.79	53.16	20.47	18.63	13.97
N. of PD ≥ 6 mm	0.00	0.00	19.25	14.86	5.67	3.92
% of PD ≥ 6 mm	0.00	0.00	12.46	10.44	3.68	2.66
FMPS (%)	7.45	3.39	86.84	14.34	27.39	10.48
FMBS (%)	7.36	2.66	76.79	18.51	28.17	9.39

FMPS, full-mouth plaque score; FMBS, full-mouth bleeding score; PD, probing depth; SD, standard deviation.

**Table 2:** Salivary concentration of metabolites in HI and GP patients at baseline and 3 months post-treatment. *p*-values, adjusted *p*-values and false discovery rate (FDR) are reported for overall comparison and pairwise analyses.

Metabolites	Generalized Periodontitis (GP) Patients				Healthy Individuals (HI)		Kruskal–Wallis		Paired Wilcoxon		U Mann–Whitney	
	Baseline (B)		After Treatment (AT)				B, AT and HI		B vs. AT		HI vs. AT	
	Mean	SD	Mean	SD	Mean	SD	*p*-Value	FDR	*p*-Value	FDR	*p*-Value	FDR
alanine	2318.68	2156.2	835.16	400.38	731.26	324.31	0.099	0.162	0.052	0.180	0.740	0.882
acetate	82,191.52	63,733.79	119,495.6	38,614.2	180,849.4	63,915.1	**0.002**	**0.033**	0.176	0.304	**0.004**	0.058
propionate	10,620.21	8802.07	18,185.02	14,457.12	16,615.03	9427.33	0.327	0.406	0.204	0.316	0.880	0.909
glycine	4896.88	3390.89	4134.86	1717.69	3252.59	1542.33	0.446	0.512	0.470	0.560	0.347	0.598
butyrate	460.82	573.65	811.82	726.82	540.03	563.16	0.187	0.259	0.151	0.293	0.288	0.524
taurine	1551.22	886.83	1053.22	447.67	946.69	452.63	0.089	0.154	0.151	0.293	0.782	0.892
ethanol	170.8	126.63	294	248.31	529.93	325.07	**0.003**	**0.033**	0.204	0.316	**0.037**	0.129
proline	389.02	498.62	363.87	218.22	176	122.96	0.076	0.146	0.910	0.970	**0.016**	0.081
succinate	1270.54	2849.59	1783.53	1496.6	3052.11	3424.66	**0.022**	0.087	0.129	0.293	0.651	0.861
leucine	1639.75	1527.77	689.34	441.56	697.94	171.73	**0.035**	0.102	**0.042**	0.180	0.230	0.509
valine	1077.09	1001.68	305.23	335.29	551.16	245.92	**0.005**	**0.036**	**0.001**	**0.045**	**0.009**	0.066
isovalerate	479.45	450.54	536.68	453.33	438.91	235.4	0.868	0.897	0.910	0.970	0.805	0.892
isocaproate	148.83	168.95	222.04	144.81	315.28	274.43	0.162	0.239	0.151	0.293	0.566	0.835
acetoin	201.97	167.61	210.34	165.32	510.06	283.17	**0.002**	**0.033**	0.910	0.970	**0.002**	0.058
tyrosine	509.97	456.14	307.53	210.88	270.99	232.4	0.472	0.522	0.129	0.293	0.442	0.720
methylamine	298.9	354.4	169.15	73.67	274.34	96.61	0.064	0.140	0.301	0.413	**0.006**	0.058
phenylalanine	1177.75	1088.23	479.24	169.59	547.29	188.64	**0.036**	0.102	**0.034**	0.177	0.566	0.835
isoleucine	636.39	714.79	143.31	98.65	193.49	79.1	**0.012**	0.063	**0.007**	0.071	0.104	0.292
galactose	80.22	93.82	140.2	166.6	139.57	285.92	0.738	0.789	0.176	0.304	0.644	0.861
lactate	3359.7	2674.72	5413.35	5361.44	1349.14	1394.03	**0.019**	0.085	0.470	0.560	**0.011**	0.066
aspartate	155.65	104.89	106.12	94.69	58.25	37.36	0.068	0.140	0.129	0.293	0.185	0.442
creatine	295.84	307.4	226.62	413.65	494.94	558.86	0.080	0.146	0.301	0.413	**0.021**	0.087
choline	848.12	834.16	879.44	908.5	1372.24	526.06	**0.041**	0.107	0.970	0.970	**0.023**	0.087
methanol	564.8	343	1452.51	2117.43	637.3	739.04	0.134	0.208	0.052	0.180	0.079	0.246
pyruvate	470.4	460.62	961.51	731.33	1077.92	1722.88	0.192	0.259	0.129	0.293	0.288	0.524
formate	776.5	1976.83	1656.13	2726.19	513.89	799.4	0.206	0.266	**0.016**	0.100	0.176	0.442
glucose	161.94	188.36	74.34	83.34	70.2	97.73	0.365	0.435	0.380	0.491	0.667	0.861
sarcosine	253.02	279.33	244.6	257.13	202.9	75.21	0.980	0.980	0.970	0.970	0.878	0.909
hypoxanthine	167	303.72	33.43	20.06	30.65	20.27	**0.010**	0.059	**0.012**	0.095	0.740	0.882
uracil	383.07	622.49	61.67	49.7	74.81	65.7	**0.030**	0.102	**0.005**	0.071	1.000	1.000
isopropanol	181.78	134.02	293.53	221.85	406.78	247.21	**0.048**	0.114	0.307	0.413	0.260	0.524
